# The C-terminal proline-rich repeats of Enteropathogenic *E. coli* effector EspF are sufficient for the depletion of tight junction membrane proteins and interactions with early and recycling endosomes

**DOI:** 10.1186/s13099-024-00626-8

**Published:** 2024-07-07

**Authors:** Imran Ansari, Anupam Mandal, Kritika Kansal, Pangertoshi Walling, Sumbul Khan, Saima Aijaz

**Affiliations:** Special Centre for Molecular Medicine, Jawaharlal Nehru University, New Delhi, 110067 India

**Keywords:** Enteropathogenic *E. Coli*, EspF, Intestinal barrier, Proline rich repeats, Tight Junctions, Endocytosis

## Abstract

**Background:**

Enteropathogenic *E. coli* (EPEC) causes acute infantile diarrhea accounting for significant morbidity and mortality in developing countries. EPEC uses a type three secretion system to translocate more than twenty effectors into the host intestinal cells. At least four of these effectors, namely EspF, Map, EspG1/G2 and NleA, are reported to disrupt the intestinal tight junction barrier. We have reported earlier that the expression of EspF and Map in MDCK cells causes the depletion of the TJ membrane proteins and compromises the integrity of the intestinal barrier. In the present study, we have examined the role of the proline-rich repeats (PRRs) within the C-terminus of EspF in the depletion of the tight junction membrane proteins and identified key endocytosis markers that interact with EspF via these repeats.

**Results:**

We generated mutant EspF proteins which lacked one or more proline-rich repeats (PRRs) from the N-terminus of EspF and examined the effect of their expression on the cellular localization of tight junction membrane proteins. In lysates derived from cells expressing the mutant EspF proteins, we found that the C-terminal PRRs of EspF are sufficient to cause the depletion of TJ membrane proteins. Pull-down assays revealed that the PRRs mediate interactions with the TJ adaptor proteins ZO-1 and ZO-2 as well as with the proteins involved in endocytosis such as caveolin-1, Rab5A and Rab11.

**Conclusions:**

Our study demonstrates the direct role of the proline-rich repeats of EspF in the depletion of the TJ membrane proteins and a possible involvement of the PRRs in the endocytosis of host proteins. New therapeutic strategies can target these PRR domains to prevent intestinal barrier dysfunction in EPEC infections.

**Supplementary Information:**

The online version contains supplementary material available at 10.1186/s13099-024-00626-8.

## Background

Infections by EPEC are one of the leading causes of infantile diarrhea in the developing world [1, 2]. EPEC uses a type III secretion system to transfer at least 20 effector proteins into the host intestinal cells which disrupt multiple signaling pathways [3]. Some of these effectors cause an increase in permeability across the intestinal barrier. This barrier is composed of epithelial cells that are attached to each other through tight junctions (TJs) [4, 5]. The transmembrane proteins of the TJs, namely claudins and occludin, form a selective semi-permeable barrier to regulate the passage of charged ions and uncharged molecules, respectively [4, 5]. At their C-terminus, these transmembrane proteins contain binding sites for the cytoplasmic adaptor proteins such as the zonula occludens (ZO) proteins which in turn interact with the actin cytoskeleton [4, 5]. The ZO proteins also regulate permeability across TJs by controlling actin contractility [4, 5].

Pathogens such as EPEC disrupt the intestinal barrier by displacing the TJ transmembrane proteins from the plasma membrane and increasing the flux of ions and solutes through the host intestinal barrier [3, 6]. EPEC translocates more than twenty effectors into the host cells of which EspF, EspG1/G2, Map and NleA are reported to disrupt the intestinal TJ barrier [6–8]. Experiments conducted on mice and cultured HeLa, Caco-2 and T84 cells infected with EPEC have identified some of the processes involved in the disruption of TJs [9, 10]. These studies have shown that EPEC perturbs the barrier by the EspF-mediated removal of occludin and ZO-1 from the TJs, a decrease in transepithelial resistance and an increase in the permeability of electrolytes [11–14]. Subsequently, another EPEC effector, Map, was reported to disrupt TJs independent of EspF [12, 15]. We have shown earlier that EspF and Map not only displace the TJ proteins from the plasma membrane into the cytoplasm but also deplete the total levels of TJ membrane proteins [16]. While partial recovery was observed in the levels of claudin-4 and occludin by the addition of chloroquine, suggesting a possible involvement of the lysosomes in the degradation of these proteins, no recovery was observed for claudin-1 which is an important regulator of ion permeability in the intestine [16]. EspF had a more potent effect than Map on TJ disruption with cell lines expressing EspF showing a significant reduction in the transcript levels of *claudin-1* and the total protein levels of claudin-1, claudin-4 and occludin [16]. These effects were independent of the role of EspF in mitochondrial dysfunction implying that the N-terminal mitochondrial targeting signal (MTS) of EspF is not involved in TJ disruption [16]. EspF derived from EPEC is a 206 amino acid protein that contains three proline-rich repeats (PRRs) at the C-terminus [17]. Each PRR module contains a sorting nexin 9 (SNX9) binding site at the N-terminus consisting of conserved residues RxAPxxP and an N-WASP (neuronal Wiskott-Aldrich syndrome protein) binding domain at the C-terminus [17]. SNX9 has a role in regulating membrane curvature and its interaction with EspF promotes the formation of membrane tubules in infected cells [18]. N-WASP regulates actin polymerization by the direct recruitment and activation of the Arp2/3 complex [17, 18]. These interactions allow EspF to coordinate both host plasma membrane alterations as well as actin polymerization. Current data suggests a role of the SNX9 binding site in the endocytosis of membrane proteins. However, as reported by us earlier [16], the disruption of the TJ barrier is caused not only by the displacement of the TJ membrane proteins from the plasma membrane but also their eventual depletion. We therefore examined if the PRR modules of EspF are involved in the depletion of the TJ membrane proteins. Here we report that the proline-rich repeats (PRRs) at the C-terminus of EspF cause the depletion of the TJ transmembrane proteins and also mediate interactions of EspF with caveolin-1, Rab5A, Rab11 and the TJ adaptor proteins ZO-1 and ZO-2.

## Methods

### Generation of EspF deletion constructs

EspF deletion mutants (PRR-1-2-3, PRR-2-3, PRR-3 and PRR-1-2) were generated by PCR using genomic DNA derived from Enteropathogenic *Escherichia coli* O127:H6 strain E2348/69 and forward and reverse primers listed below.

EspF (F): 5’-AAAAAGGATCCCTTAAGATGGTTAATGGAATTAGTAACGCTG-3’.

EspF-PRR-1-2-3 (F): 5’-AAAAAGGATCCCTTAAGATGGCTCGTCCGGCACCGCCGCCA-3’.

EspF-PRR-2-3 (F): 5’-AAAAAGGATCCCTTAAGATGGCCCGTCCGGCACCGCCGCCA-3’.

EspF-PRR-3 (F): 5’-AAAAAGGATCCCTTAAGATGGCCCGTCAGGCACCACCGCC-3’.

EspF-PRR-1-2 (R): 5’- AAAAATCTAGAGTCGACTGGCTTAAAGCTTACAGTCTC-3’.

EspF (R): 5’-AAAAATCTAGAGTCGACCCCTTTCTTCGATTGCTCATAGG-3’.

The PCR fragments were cloned in pAcGFP1-C1 vector between the *Bgl*II and *Sal*I sites (for N-terminal GFP tag) and pGEX-4T-3 vector between the *Bam*HI and *Sal*I sites (for N-terminal GST tag).

### Cell culture

MDCK-II cells were grown in DMEM supplemented with 10% FBS and 1X penicillin-streptomycin solution at 37 °C in a CO_2_ incubator. Stable cell lines were generated by following the calcium chloride precipitation method. Briefly, MDCK cells were grown in DMEM medium without antibiotics in 6 well plates until they were 40% confluent. Transfections were carried out by mixing 2–5 µg of plasmid DNA in 250 µl of 2X HEPES buffer (pH 7.1) with 250 µl of freshly prepared 0.25 M CaCl_2_ solution for 30 min at room temperature. This mixture was added to the wells containing MDCK cells and the plates were incubated at 37 °C for 20 min after which 1 ml of warm medium was added and the plates incubated overnight. The next day, the medium was removed and the cells were incubated with 12.5% sterile glycerol in DMEM medium for 2 min at room temperature. The glycerol solution was removed and the cells were rinsed twice with PBS following which DMEM medium containing 10% FBS was added. After 24 h, the selection medium containing 500 µg/ml G418 was added. After 24 h in selection medium, the cells were trypsinized and grown in 100 mm culture plates for 3 weeks after which individual clones were picked and checked for the expression of GFP-tagged proteins by Western blotting. At least 25–30 independent clones were picked for each cell line and analyzed for the expression of GFP-tagged proteins of appropriate molecular weights. The expression of GFP-tagged proteins was confirmed in at least 10 clones for each cell line. From these confirmed clones, a minimum of 3 clones were used in each experiment.

### Pull-down assays

BL21(DE3)pLysS cells were transformed with pGEX-4T-3 vector and GST-tagged EspF deletion constructs using standard protocols. Protein expression was induced with 1 mM IPTG at 37 °C for 6 h. The cultures were centrifuged and the pellet resuspended in lysis buffer (1X PBS, 0.5% Triton X-100, 1 mM DTT and 1 mM PMSF) followed by sonication and centrifugation at 12,000 rpm for 15 min at 4 °C. The supernatants were used in pull-down assays. Briefly, 20 µg of each recombinant protein was immobilized on Glutathione sepharose beads overnight at 4 °C followed by washing with lysis buffer containing 1% Triton X-100. MDCK cell extracts were prepared by resuspending the cells grown on a 100 mm culture plate in cell lysis buffer containing 1% Triton X-100 and extracted by passing through a 23-gauge needle. The cell extract was incubated on ice for 30 min and then centrifuged at 12,000 rpm for 10 min at 4 °C. The supernatant was added to the Glutathione sepharose beads containing immobilized GST-tagged proteins and mixed on a rotator overnight at 4 °C. The bead-bound complexes were washed with cell lysis buffer containing 0.5% Triton X-100 and analyzed by Western blotting.

### Immunofluorescence assays and microscopy

MDCK cells and stable cell lines expressing either the GFP vector or different EspF constructs, grown on coverslips, were fixed with chilled methanol for 5 min at -20 °C, rehydrated in PBS at room temperature for 5 min followed by blocking with PBS containing 0.5% BSA. The coverslips were incubated with primary antibodies diluted in PBS containing 0.5% BSA for 4 h at room temperature, washed and incubated with anti-mouse or anti-rabbit Cy3-conjugated secondary antibodies (Merck) for 1 h at room temperature. Primary antibodies were purchased from Thermo Fisher Scientific (claudin-1, claudin-4, occludin, ZO-1, ZO-2, Caveolin-1, Rab11, Rab5A, GFP) and Cell Signaling Technologies (actin). Images were acquired at 100X magnification on an ApoTome microscope (Zeiss, Axiovert 40 CFL) using the image acquisition software ZEN (version 2.3, Blue Edition). Brightness and contrast were adjusted for whole images. For making the figures, whole images were used after resizing.

### Preparation of total protein lysates

Total protein lysates were prepared from confluent MDCK cells and stable cell lines expressing GFP vector or the EspF deletion constructs grown on culture plates, by adding Laemelli buffer and extracting through a 23-gauge needle several times followed by Western blotting using the antibodies described above. GAPDH was used as a loading control. The band intensities were quantitated using ImageJ software (https://imagej.nih.gov/ij) and plotted with respect to MDCK cells (normalized to 1). Comparisons were made between lysates derived from 3 independent cell lines for each construct and experiments were performed at least three times.

### Statistical analysis

Data was analyzed using one-way ANOVA and p-values < 0.05 were considered significant.

## Results

### The PRR domains of EspF are sufficient for the depletion of TJ proteins

**Fig. 1:**
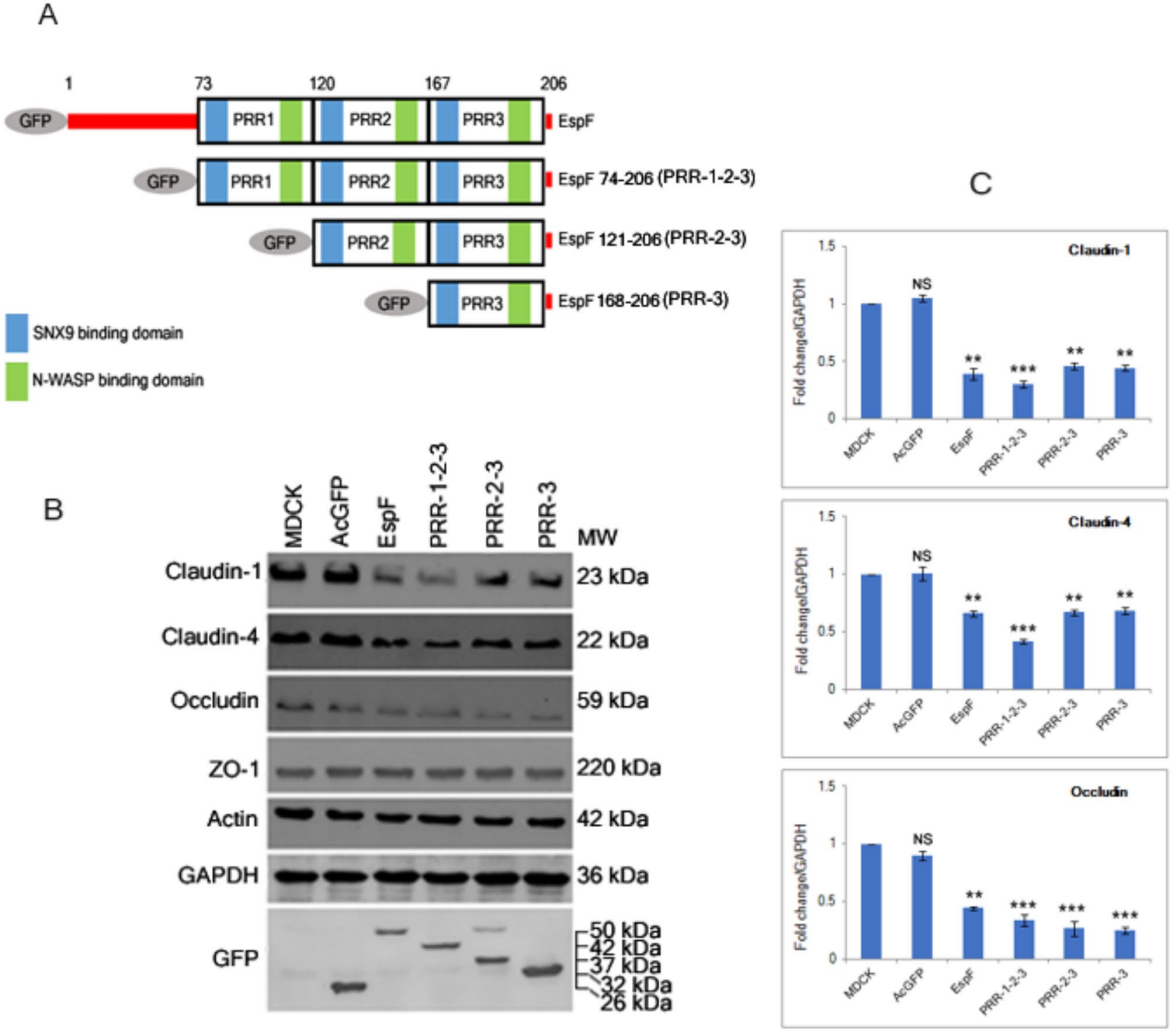
Expression of PRR domains of EspF depletes the total levels of TJ proteins. **(A)** Schematic representation of mutant EspF constructs. **(B**) Cell lysates obtained from wild type MDCK cells or stable cells lines expressing GFP vector (AcGFP), GFP-EspF (EspF), GFP-PRR-1-2-3 (containing all three PRRs), GFP-PRR-2-3 (containing PRR-2-3) and GFP-PRR-3 (containing only PRR-3) were analyzed by Western blotting. **(C)** Band intensities were measured by ImageJ software and fold change in the expression of each protein with respect to MDCK cells (normalized to 1) was plotted relative to GAPDH. Three cell lines were analyzed for each EspF construct and experiments were performed at least three times. A representative blot from one experiment is shown. Bars represent means ± s.e.m from three independent experiments; **p value < 0.005 and ***p value < 0.0005. No change was seen in the levels of the TJ adaptor ZO-1 or actin (not shown in panel **C**)

We generated N-terminally GFP-tagged EspF constructs that progressively lacked the N-terminal regions of EspF. These constructs were transfected into MDCK cells to generate stable cell lines constitutively expressing the mutant EspF proteins. Total cell lysates were prepared from these cell lines to confirm the expression of proteins of ~ 50 kDa (GFP-EspF), ~ 42 kDa (GFP-PRR-1-2-3), ~ 37 kDa (GFP-PRR-2-3) and ~ 32 kDa (GFP-PRR-3). Our previous data has shown that EspF depletes the total levels of TJ proteins and the N-terminal mitochondrial targeting signal of EspF is not involved in TJ disruption [16]. Thus, we checked if the C-terminal PRR modules of EspF are responsible for this depletion. Cell lysates derived from confluent cell lines expressing GFP vector alone, full length GFP-EspF or cell lines expressing all three PRRs (GFP-PRR-1-2-3), two PRRs (GFP-PRR-2-3) or only the last PRR (GFP-PRR-3) were analyzed by Western blotting using antibodies against TJ proteins. All stable cell lines expressing the mutant EspF proteins exhibited a significant decrease in the levels of claudin-1, claudin-4 and occludin while no change was seen in the levels of the TJ adaptor protein, ZO-1 (Fig. 1). The expression of claudin-1 was reduced to ~ 0.39, ~ 0.30, ~ 0.46 and ~ 0.44 fold, respectively in GFP-EspF, GFP-PRR-1-2-3, GFP-PRR-2-3 and GFP-PRR-3 cell lines as compared to MDCK cells. The expression level of claudin-4 was reduced to ~ 0.66, ~ 0.42, ~ 0.67, and ~ 0.68 fold, respectively in GFP-EspF, GFP-PRR-1-2-3, GFP-PRR-2-3 and GFP-PRR-3 stable cell lines. The expression of occludin was reduced to ~ 0.44, ~ 0.34, ~ 0.27 and ~ 0.25 fold, respectively in cells expressing GFP-EspF, GFP-PRR-1-2-3, GFP-PRR-2-3 and GFP-PRR-3 as compared to MDCK cells. These data indicate that the C-terminal PRRs of EspF regulate the depletion of TJ membrane proteins and a single PRR domain is sufficient to deplete the total levels of claudin-1, claudin-4 and occludin (Fig. 1). We did not observe any depletion in the total levels of ZO-1 or actin suggesting that EspF specifically targets the TJ membrane proteins for depletion.

### EspF-PRR domains dislocate TJ membrane proteins from the plasma membrane

**Fig. 2:**
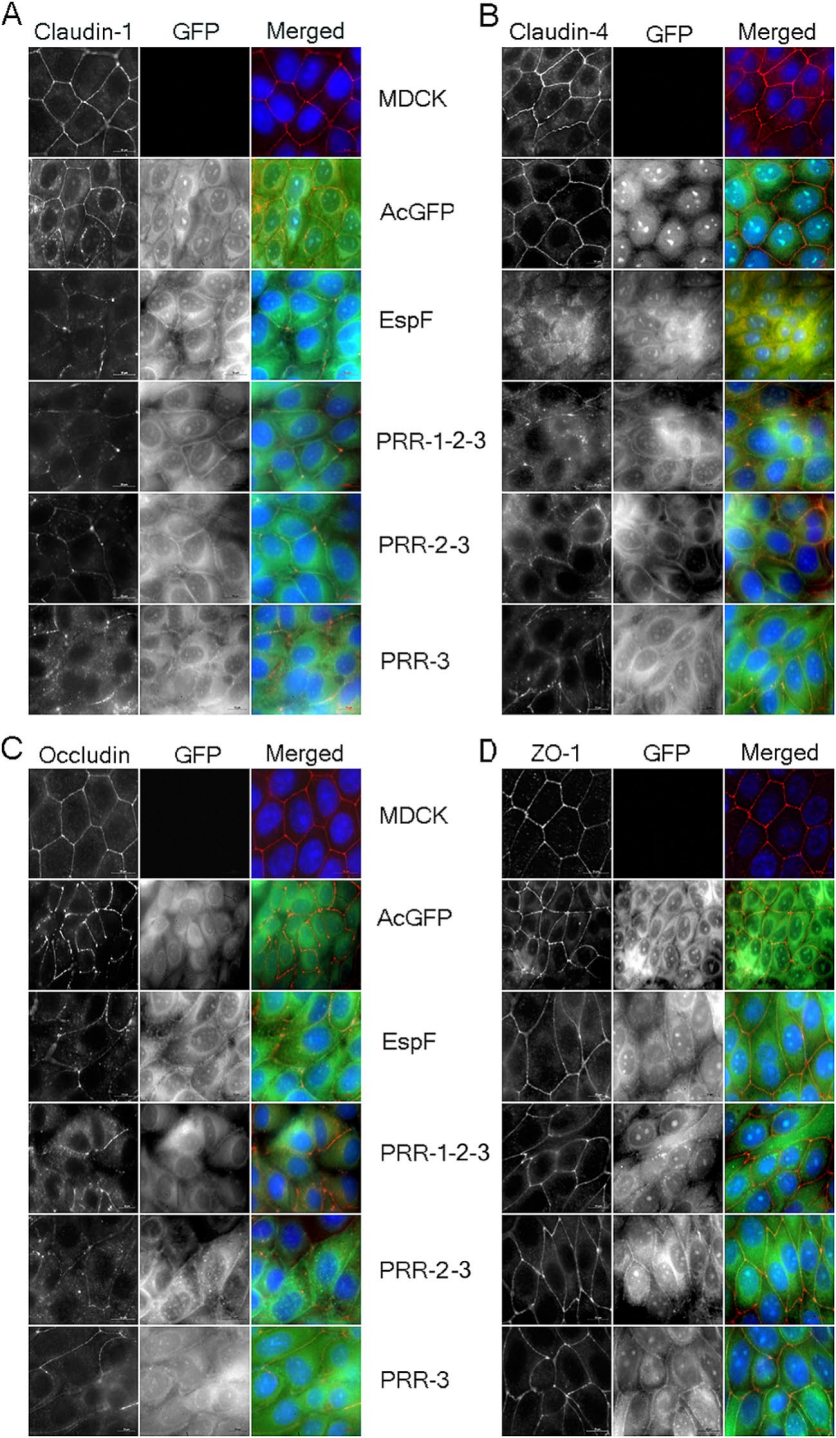
Expression of the EspF PRR domains affects the cellular localization of claudin-1, claudin-4 and occludin. Stable cell lines expressing GFP vector alone, GFP-EspF or GFP-tagged constructs containing one or more EspF-PRR domains were grown on coverslips and the cells were labeled with antibodies against claudin-1 **(A)**, claudin-4 **(B)**, occludin **(C)** and ZO-1 **(D)**. The localization pattern of TJ proteins were examined by microscopy. Scale bars: 10 μm. TJ proteins are in red; nucleus is shown in blue

Next, we examined the cellular localization of claudin-1, claudin-4, occludin and ZO-1 in confluent monolayers of MDCK cells and stable cell lines expressing GFP vector, GFP-EspF or GFP-PRR-1-2-3, GFP-PRR-2-3 or GFP-PRR-3 by immunocytochemistry. Cells were grown on coverslips until confluent, fixed and labeled with respective TJ antibodies. As seen for full length GFP-EspF, all three truncated proteins expressing different PRRs were found to be localized mostly in the cytoplasm and to a lesser extent at the plasma membrane. The expression of truncated EspF proteins caused the delocalization of claudin-1, claudin-4 and occludin from the apical junctional complex to the cytoplasm while ZO-1 remained at the junctional complex (Fig. 2). The delocalization of the TJ proteins was more pronounced in the cytoplasm of stable cell lines expressing GFP-PRR-1-2-3, GFP-PRR-2-3 and GFP-PRR-3 as compared to cell lines expressing wild type GFP-EspF.

### EspF-PRR domains interact with the TJ adaptors proteins ZO-1 and ZO-2

**Fig. 3:**
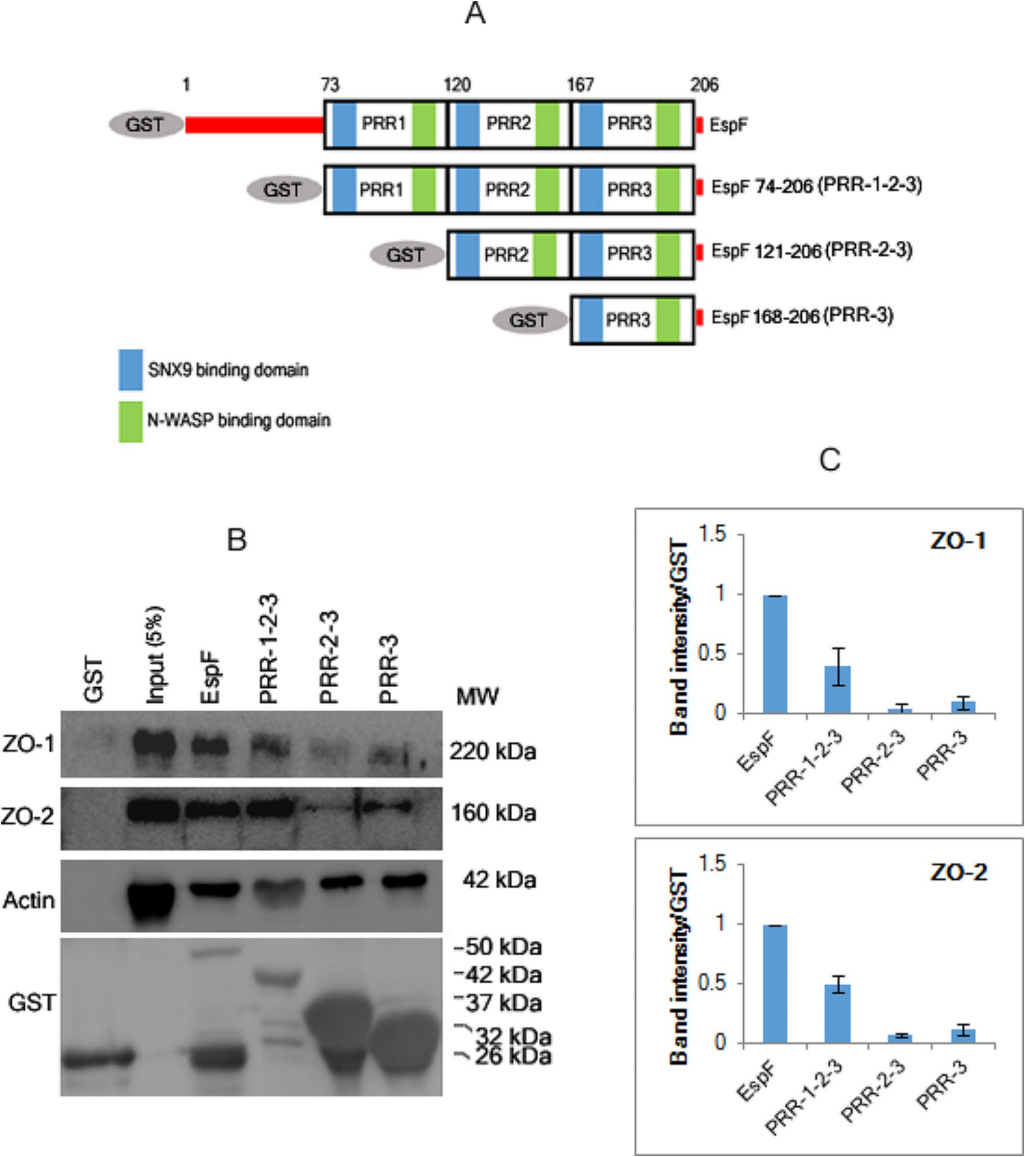
The EspF PRR domains mediate the interaction of EspF with the TJ adaptor proteins ZO-1 and ZO-2. **(A)** Schematic representation of mutant GST-tagged EspF constructs. **(B)** Pull-down assays using GST, GST-EspF or different GST-tagged constructs carrying one or more PRR domains were performed to show that EspF interacts with the TJ adaptor proteins ZO-1 and ZO-2 through the PRR domains. **(C)** The efficiency of the pull down products was quantified by calculating the band intensities relative to the level of expression of the corresponding GST-tagged EspF mutant proteins. Comparisons were made with the pull down efficiency seen in full length GST-EspF protein (normalized to 1) and graphs were plotted

We have shown earlier that EspF forms a complex with ZO-1 but not with occludin, claudin-1 or claudin-4 [16]. To determine if this interaction was mediated by the PRR domains of EspF, the PCR amplified fragments described in materials and methods section were cloned in pGEX-4T-3 vector to obtain N-terminal GST-tagged recombinant EspF truncated proteins containing either all 3 PRRs (GST-PRR-1-2-3), 2 PRRs (GST-PRR-2-3) or only the last PRR (GST-PRR-3). In addition, we also generated a GST-tagged construct of EspF where only the last PRR was deleted (construct called GST-PRR-1-2). These recombinant proteins were used in pull-down assays to examine their interaction with ZO-1 (Fig. 3). The specificity of the pull-down reactions was first confirmed by probing the blots with anti-actin antibody because all the three PRR modules of EspF have been shown to interact with actin [16, 17]. EspF mutant proteins containing all three PRR domains were found to interact with ZO-1. Additionally, we also found ZO-2 in the pull-down complex with GST-EspF, GST-PRR-1-2-3, GST-PRR-2-3 and GST-PRR-3 mutant proteins (Fig. 3). The efficiency of the pull down of ZO-1 and ZO-2 by each of the GST-tagged EspF mutant proteins was estimated by quantifying the band intensities of ZO-1 and ZO-2 in the western blots with respect to the expression levels of each GST-tagged EspF truncated protein. Comparisons were made with the pull down efficiency observed with full length GST-EspF protein (normalized to 1) and bar graphs were plotted (Fig. 3C). As shown in Fig. 3, the efficiency of the ZO-1 and ZO-2 pull down decreased in reactions performed with GST-PRR-2-3 and GST-PRR-3.

### The PRR domains of EspF interact with caveolin-1, Rab5A and Rab11

**Fig. 4:**
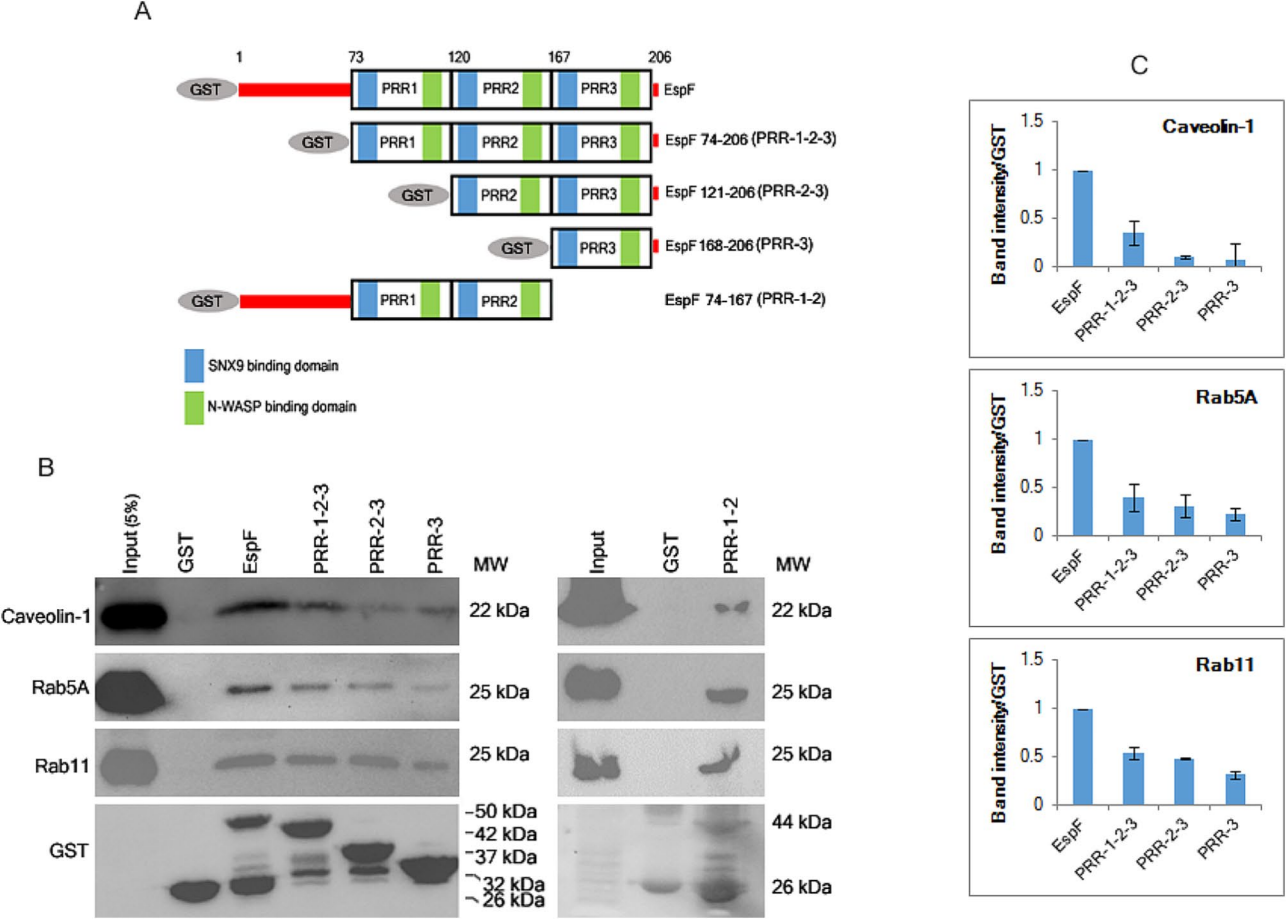
The EspF PRR domains mediate the interaction of EspF with Caveolin-1, Rab5A and Rab11. **(A)** Schematic representation of GST-tagged EspF, GST-PRR-1-2-3, GST-PRR-2-3, GST-PRR-3 and GST-PRR1-2 constructs. **(B)** Pull-down assays using GST-EspF or different GST-tagged constructs carrying one or more PRR domains were performed to show that EspF interacts with Caveolin-1, Rab5a and Rab11 through the PRR domains. **(C)** Pull down efficiency of the different PRR proteins was compared with that of full length EspF (normalized to 1) and graphs were plotted

EspF has been reported to regulate the endocytosis of plasma membrane proteins in a SNX9-dependent manner [18]. Therefore, we examined whether EspF interacts with the host endocytosis machinery to cause the trafficking of TJ transmembrane proteins from the plasma membrane into the cytoplasm. TJs are dynamic complexes whose turnover is regulated by the internalization of existing proteins by endocytosis and their replacement with new proteins [19]. Endocytosis of TJ proteins may involve the clathrin-dependent pathway or the caveolin-mediated pathway [20, 21]. In epithelial cells such as MDCK, actin depolymerization has been reported to cause the endocytosis of claudin-1 and occludin by caveolin-mediated mechanisms [21]. To examine if EspF interacts with caveolin-1, we performed pull-down assays using GST-tagged proteins expressing different PRR domains. Full length EspF and all the PRR mutant proteins were found to interact with caveolin-1. However, when the band intensities of caveolin-1 in each reaction were quantified relative to the expression levels of the corresponding GST-tagged EspF truncated proteins, the pull down efficiency of caveolin-1 was found to decrease in reactions performed with GST-PRR-2-3 and GST-PRR-3 proteins (Fig. 4). After internalization, the plasma membrane proteins are sorted into endosomes which may recycle them back to the plasma membrane or target them for degradation. Endosomes are classified into early, late and recycling endosomes based on the stages of internalization, morphology, lipid composition and expression of Rab GTPases. Markers for early, late and recycling endosomes include Rab5A, Rab7, and Rab11 respectively [22]. We examined if the PRR domains of EspF interact with Rab5A and Rab11. Pull-down assays performed using full length GST-EspF, GST-PRR-1-2-3, GST-PRR-2-3 and GST-PRR-3 proteins showed that all the PRR domains of EspF interact with Rab5A and Rab11 (Fig. 4B, C). We also performed pull down assays with a GST-tagged EspF protein lacking the PRR-3 module (GST-PRR-1-2) and found that this truncated protein also interacted with caveolin-1, Rab5A and Rab11. This suggests that regions within each PRR domain mediate binding of caveolin-1, Rab5A and Rab11.

Our immunocytochemistry data showed the co-localization of caveolin-1 vesicles with EspF at the plasma membrane (Supplementary Fig. 1). Notably, the colocalization of EspF with caveolin-1 was more pronounced in the sub-apical region as indicated by a broader area of colocalization at the plasma membrane as compared to caveolin-1 localization in cells expressing the GFP vector alone. No change was observed in the localization of clathrin vesicles in cells expressing EspF nor did we find an interaction of EspF with clathrin (data not shown). Alto et al., [18] have reported that EspF transiently interacts with clathrin at the plasma membrane in a spatio-temporal manner so it is possible that we may have missed this interaction. EspF also co-localized with markers of early (Rab5A) and recycling (Rab11) endosomes in cells expressing GFP-tagged EspF and control cells expressing GFP vector alone. Rab5A was observed to be distributed throughout the cytoplasm in controls cells expressing GFP vector alone. However, in stable cell lines expressing EspF, Rab5A colocalized with EspF at the plasma membrane as well as in the cytoplasm (Supplementary Fig. 1). In control cells expressing GFP vector alone, Rab11 was distributed in the cytoplasm with vesicles extending to the cell periphery with a more distinct concentration seen in the perinuclear region. In cells expressing GFP-EspF, Rab11 localization was mostly seen in the perinuclear region (Supplementary Fig. 1).

## Discussion

EPEC infections cause acute infantile diarrhea leading to dehydration and death [1, 2]. One of the underlying causes of dehydration is the excessive leakage of water and electrolytes through the intestinal tight junction barrier [23]. EPEC is an extracellular pathogen that causes infection by the translocation of at least 20 effectors into the host intestinal cells through a type three secretion system. Attachment of EPEC to the apical membrane of host intestinal enterocytes is mediated by the intimate adhesion between the bacterial outer surface protein, intimin and its receptor Tir (*T*ranslocated *i*ntimin *r*eceptor) which is inserted into the plasma membrane of the host cells after translocation [3, 23]. After translocation inside the host cell, EspF inactivates the sodium-D-glucose co-transporter (SGLT-1), inhibits Na^+^/H^+^ exchanger 3 (NHE3), internalizes aquaporins-2/-3 and disrupts the TJs causing severe disruption of the host cell functions [12, 17, 24]. At its N-terminus, EspF from EPEC contains a mitochondrial targeting signal (residues 1–24) and a nucleolar targeting domain (residues 21–74) which disrupt the functions of these organelles [17]. At the C-terminus, EspF has three proline rich repeats (PRRs) each of which contain binding sites for SNX9 and N-WASP [17, 18]. EspF derived from the rabbit EPEC strain, E22, binds actin and recruits the TJ adaptor proteins ZO-1 and ZO-2 into the actin pedestals [25]. EspF is reported to cause the caspase-dependent cleavage and subsequent loss of EGFR in intestinal epithelial cells [26]. EspF also causes the endocytosis of the polarity determining protein Crb3 in a SNX9-dependent manner and the redistribution of the apical Na^+^/K^+^ ATPase [27].

Several studies have shown that EspF disrupts the intestinal barrier by decreasing transepithelial electrical resistance, thus increasing the permeability of charged ions and uncharged molecules [6, 12, 13, 17]. Additionally, we reported earlier that MDCK cells stably expressing EspF caused a reduction in the transcripts of *claudin-1* and *occludin* and depleted the protein levels of claudin-1, claudin-4 and occludin [16]. EspF expression also caused the dislocation of the existing claudin-1, claudin-4 and occludin from the plasma membrane [16]. Another study has shown that the infection of cells with EPEC or *Citrobacter rodentium* carrying a mutant version of EspF, lacking the N-WASP and SNX9 binding sites within each PRR module, did not induce TJ disruption [28]. This study also suggested that binding of N-WASP and SNX9 with EspF mediates the re-localization of the TJ protein ZO-1. A more recent study examined the role of different EspF domains in the modulation of host lysosomes and showed that EspF causes the secretion of lysosomal enzymes into the extracellular medium [29]. In this study, MDCK and HeLa cells were infected with strains of EPEC carrying mutations in the SNX9 and N-WASP binding sites within each PRR domain of EspF [29]. This study concluded that these EspF domains were not involved in the secretion of lysosomal enzymes [29]. Notably, the secretion of lysosomal enzymes was found to be similar in HeLa, CaCo-2 BBe and MDCK cells infected with EPEC [29] suggesting a common mechanism used by EPEC in these different cell types. However, the role of the mutant PRR domains of EspF on the disruption of TJs was not examined in this study [29]. The involvement of EspF in the recruitment of early and recycling endosomes to the apical plasma membrane has been demonstrated in polarized MDCK cells [30] where EspF (together with the EPEC effector Map) remodels the endosomes and causes the trafficking of transferrin receptors, β1 integrins and aquaporins to the sites of infection at the plasma membrane [30]. The SNX9-dependent endocytosis of the polarity determining protein Crb3 by EspF has also been demonstrated [27]. Thus EspF plays a major role in modulating the host endocytosis pathways. Whether the disruption of TJs by EspF is caused by the endocytosis of the junctional membrane proteins has not been examined yet. Significantly, a majority of the studies examining the EPEC-induced disruption of the TJs have focused on the displacement of TJ proteins as the cause of disruption. However, we have shown earlier that TJ disruption is caused not only by the displacement of TJ proteins from the plasma membrane but also the depletion of the total levels of these proteins [16]. We now show that this effect is mediated by the PRR domains of EspF.

EspF has been reported to interact with SNX9, N-WASP, cytokeratin 18, actin, 14-3-3z, Arp2/3, profilin, ZO-1/-2 and Abcf2 [6, 16, 17, 23]. Our data shows that EspF interacts with ZO-1 via its PRR domains and this complex also includes ZO-2. Depolymerization of actin is reported to induce the caveolin-mediated endocytosis of TJ proteins [21]. As EspF also causes the reorganization of the actin cytoskeleton, we examined if EspF interacts with caveolin-1. EspF was found to interact with caveolin-1 through its PRR domains. Earlier studies have shown that EPEC modulates the functions of Rab5A and Rab11. As mentioned above, EspF causes the transcytosis of transferrin (Tfn) receptor from the basolateral surface to the apical surface in EPEC-infected polarized MDCK cells in a Rab11-dependent manner with EspF mutants lacking the binding sites for SNX9 and N-WASP showing decreased Tfn endocytosis [27, 29]. The same study also identified the interaction of EspF with SNX18, SNX33 and WIPF1. A clathrin-dependent recruitment of Rab5 to the apical plasma membrane of host cells during early stages of infection was also demonstrated [30]. However, we did not find an interaction of EspF with clathrin. This is probably due to the transient nature of this interaction as has been reported earlier [18, 30, 31]. Our data shows that the PRR modules of EspF containing the SNX9 and N-WASP binding regions mediate not only the displacement of TJ membrane proteins but also their depletion.

## Conclusion

**Fig. 5:**
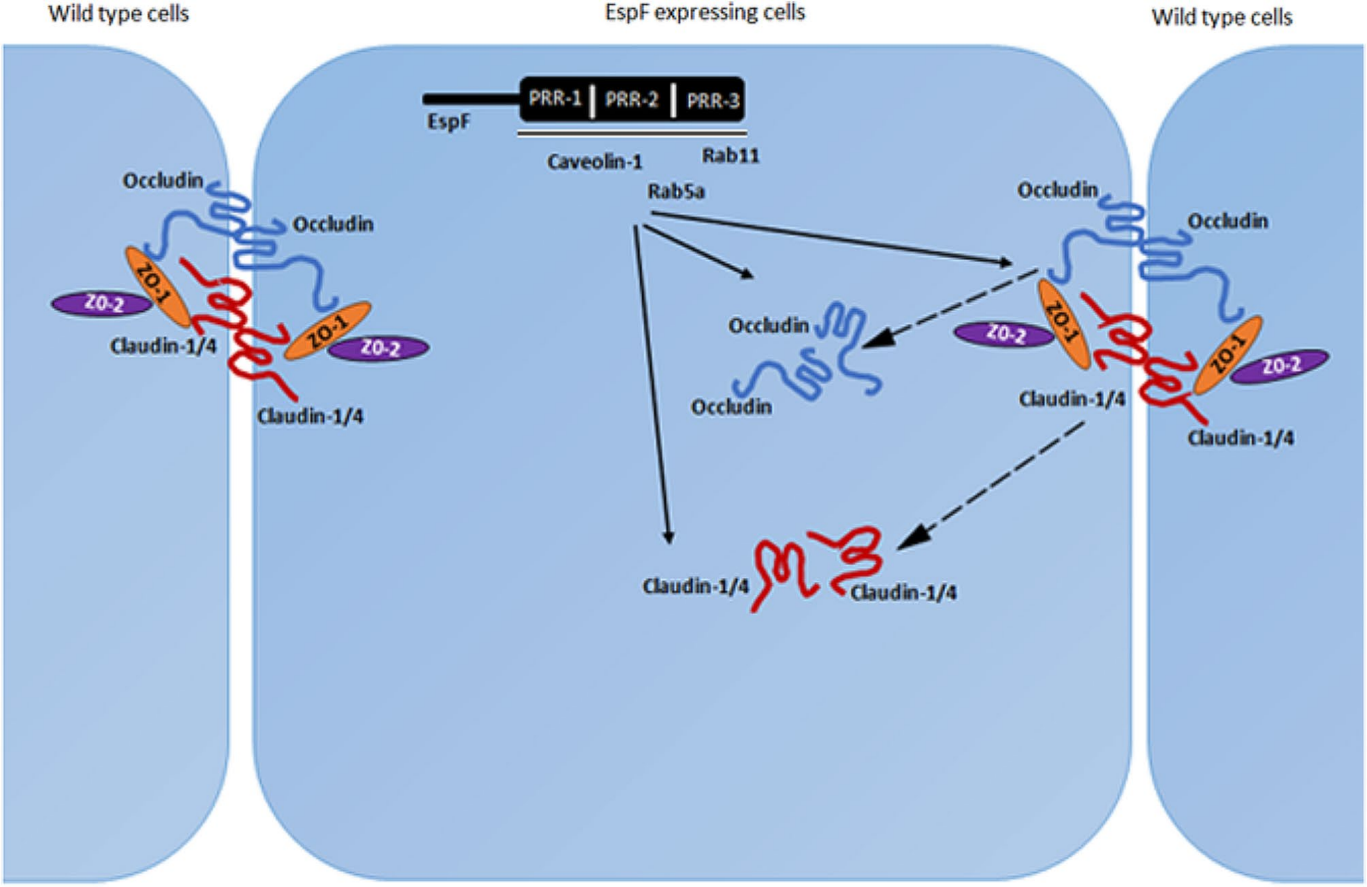
Schematic diagram showing the effect of EspF-PRR domains on tight junction membrane proteins. EspF may be recruited to the plasma membrane through its interaction with SNX9. Once at the plasma membrane, the PRR domains of EspF likely interact and activate markers of early and recycling endosomes to mediate the displacement of claudin-1, claudin-4 and occludin from the tight junctions into the cytoplasm and their depletion

Our data shows that the PRR domains of EspF mediate the depletion of the TJ proteins claudin-1, claudin-4 and occludin and regulate the interactions of EspF with markers of endocytosis such as caveolin-1, Rab5A and Rab11. Our hypothesis is that EspF interacts with SNX9 which causes its recruitment to the plasma membrane where it interacts with markers of early and recycling endosomes to internalize the TJ membrane proteins (Fig. 5). We are in the process of identifying how these PRR modules activate pathways that eventually deplete the TJ proteins. Identification of the PRR-dependent mechanisms that cause the depletion of TJ proteins will help in devising strategies to block this depletion and effectively seal the intestinal barrier in EPEC infections thus preventing the loss of infant lives.

### Electronic supplementary material

Below is the link to the electronic supplementary material.

Supplementary Material 1

## Data Availability

Data is provided within the manuscript or supplementary information files.
