# Supplementary material for: The C-terminal proline-rich repeats of Enteropathogenic E. coli effector EspF are sufficient for the depletion of tight junction membrane proteins and interactions with early and recycling endosomes
Source: Gut Pathog. 2024 Jul 7;16:36. doi: 10.1186/s13099-024-00626-8 (PMC11229284; doi:10.1186/s13099-024-00626-8)

**Supplementary Figure 1:** **EspF colocalizes with caveolin-1, Rab5A and Rab11 at the plasma membrane and the cytoplasm.** Caveolin-1, Rab5A, Rab11 are labeled in red; GFP vector and GFP-EspF are labeled in green; nucleus is labeled in blue. Scale bar: 10µm.


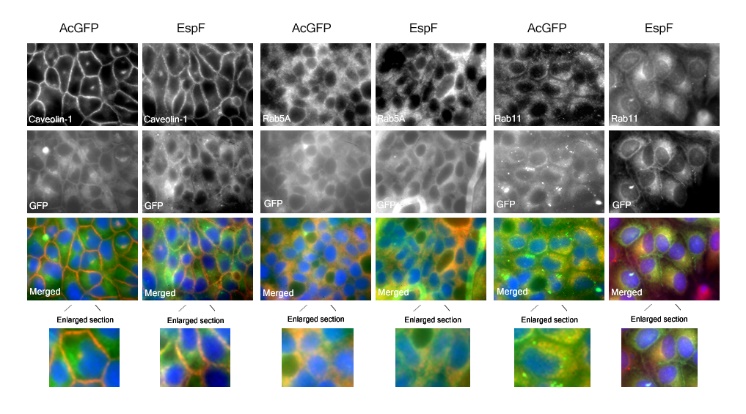

Supplement: Supplementary file 1 — Supplementary Material 1 [file 13099_2024_626_MOESM1_ESM.docx]
